# Supplementary material for: Impact of Enhanced Recovery after Surgery with Preoperative Whey Protein-Infused Carbohydrate Loading and Postoperative Early Oral Feeding among Surgical Gynecologic Cancer Patients: An Open-Labelled Randomized Controlled Trial
Source: Nutrients. 2020 Jan 20;12(1):264. doi: 10.3390/nu12010264 (PMC7019504; doi:10.3390/nu12010264)
Supplement: Supplementary file 1 [file nutrients-12-00264-s001.pdf]

Table S1. RECOvER checklist of items for reporting of ERAS compliance, outcomes and element research

	Item	Recommendation	Page
Title			
Title	1	Indicate that this is an enhanced recovery study in the title	1
Introduction			
Background	2	Explain the area of uncertainty that the study seeks to address	2
Guidelines	3	If a published set of enhanced recovery guidelines exists for this procedure, include a reference to the guidelines	3
Outcomes	4	Define the primary outcome and any key prespecified secondary outcomes for the study	5
Methods			
IRB approval	5	Give the institutional review board/ethics committee name and approval number. If permission was not required, reason should be stated	3
Study design	6	Indicate what type of study is presented. The individual guidelines for the type of study should be followed	3
Setting	7	Described whether this is a single or multicentre study and provider	3
Timing	8	Describe periods of recruitment, time points at which outcomes assessed and follow up	3
Participants	9	Define study inclusion and exclusion criteria	3
Enhanced recovery protocol	10	Describe when the enhanced recovery protocol was implemented relative to the study period (ERAS protocol initiated in November 2017)	3
	11	Provide a flow diagram or table through the continuum of care detailing the enhanced recovery protocol including the following elements:<br>a) preadmission patient education regarding the protocol<br>b) preadmission screening and optimisation as indicated for nutritional deficiency, frailty, anemia, HbA1c, tobacco cessation and ethanol use<br>c) fasting and whey protein infused carbohydrate loading<br>d) preemptive analgesia (dose, route , timing)<br>e) antiemetic prophylaxis (dose, route, timing)<br>f) intraoperative fluid management strategy<br>g) types, doses and routes of anesthetics administrated<br>h) patient warming strategy<br>i) management of post-operatiove fluids<br>j) post-operative analgesic and anti-emetic plans	Table 1

		k) plan for opioid minimization<br>l) drain and line management<br>m) early mobilization strategy<br>n) post-operative diet and bowel regimen management<br>o) criteria for discharge<br>p) tracking of post-discharge outcomes	
Enhanced recovery auditing	12	All enhanced recovery elements charted by physician assistant into enhanced recovery interative audit system	Not applicable
Outcomes	13	Primary outcomes	6
		Secondary outcomes	6
PROs	14	Patient's questionnaires	Not applicable
Results			
Patient population	15	Flow diagram to explain the deviation of study population	Figure 1
		a) Table with demographic and clinical features of study population	Table 3 & table 4
		b) Number of participants with missing data for each variable of interest	Table 5,table 6 & table 7
Enhanced recovery compliance	16	Table with average compliance for each enhanced recovery protocol element and present a comparison of the variation in enhanced recovery compliance among the study groups	Table 2
Correlations	17	Logistic regression to correlate the change in primary outcomes with study intervention	Not applicable
Discussion			
Context	18	Explain the study adds to the body of knowledge regarding the study intervention within the context of enhanced recovery after surgery care	11
Limitations	19	Discuss the limitations of the study and how these might temper the findings	12
Other information			
Funding	20	Support from departmental grant. No external grant in this study	12

*RECOvER checklist [23]. ERAS: Enhanced Recovery after Surgery.
